# Miglustat in Alzheimer's Disease Associated With Heterozygous *NPC1* Mutation: Exploratory Case Series and Preliminary Findings

**DOI:** 10.1111/ene.70419

**Published:** 2025-11-11

**Authors:** Diego Lopergolo, Daniele Gasparini, Silvia Bianchi, Barbara Pucci, Domenico Tripodi, Valerio Leoni, Andrea Chincarini, Stelvio Sestini, Henrik Zetterberg, Nicola De Stefano, Andrea Mignarri

**Affiliations:** ^1^ Department of Medicine, Surgery and Neurosciences University of Siena Siena Italy; ^2^ UOC Neurologia Azienda Ospedaliero‐Universitaria Senese Siena Italy; ^3^ Unit of Biostatistics, Epidemiology and Public Health, Department of Cardiac, Thoracic, Vascular Sciences and Public Health University of Padua Padua Italy; ^4^ Laboratory of Clinical Pathology and Toxicology, Hospital Pio XI of Desio, ASST Brianza, School of Medicine and Surgery University of Milano Bicocca Milan Italy; ^5^ Department of Science and Technology (DST) University of Sannio Benevento Italy; ^6^ National Institute of Nuclear Physics (INFN) Genoa Italy; ^7^ Department of Diagnostic Imaging, Unit of Nuclear Medicine PO‐S. Stefano, Azienda U.S.L. Toscana Centro Prato Italy; ^8^ Department of Psychiatry and Neurochemistry Institute of Neuroscience and Physiology, The Sahlgrenska Academy, University of Gothenburg Mölndal Sweden; ^9^ Clinical Neurochemistry Laboratory Sahlgrenska University Hospital Mölndal Sweden; ^10^ UK Dementia Research Institute at UCL London UK; ^11^ Department of Neurodegenerative Disease UCL Institute of Neurology London UK; ^12^ Hong Kong Center for Neurodegenerative Diseases Shatin Hong Kong SAR China; ^13^ Wisconsin Alzheimer's Disease Research Center University of Wisconsin School of Medicine and Public Health, University of Wisconsin‐Madison Madison Wisconsin USA

**Keywords:** Alzheimer's disease, brain amyloid, dementia, miglustat, *NPC1*

## Abstract

**Introduction:**

Several studies have previously demonstrated an increased risk of dementia and brain amyloid deposition in individuals with heterozygous *NPC1* mutations. Moreover, in a recent study, we identified the first family with autosomal dominant late‐onset Alzheimer's disease (AD) caused by a heterozygous *NPC1* mutation. Unfortunately, there are currently no effective treatments available for this condition. Miglustat, which impacts the metabolism of oxysterols, has been shown to exert an anti‐amyloidogenic effect in a human cellular model of AD.

**Methods:**

In our exploratory uncontrolled study, three patients from the previously published family were orally treated with miglustat for 12 months. They underwent monthly clinical evaluations and routine blood tests. Additionally, neuropsychological evaluations, brain amyloid‐PET imaging, and biochemical analyses on plasma and CSF were performed.

**Results:**

All three patients achieved clinical stability, showed a sustained reduction in serum oxysterol levels, and experienced a marked decrease in brain amyloid burden.

**Discussion:**

Based on our preliminary observations and hypothesis‐generating findings, along with the growing evidence suggesting AD as a lipid disorder, miglustat should be further tested in a larger cohort of heterozygous *NPC1* mutated patients and probably evaluated as a potential disease‐modifying treatment for AD.

## Graphical abstract

Three AD patients sharing the same heterozygous *NPC1* mutation were orally treated with miglustat for 12 months and underwent monthly clinical and neuropsychological evaluations, brain amyloid‐PET imaging, and biochemical analyses on plasma and CSF. All three patients achieved clinical stability, showed a sustained reduction in serum oxysterol levels, and experienced a marked decrease in brain amyloid burden. Based on our preliminary observations, on the light of the growing evidence suggesting AD as a lipid disorder, miglustat should be further tested in a larger cohort of heterozygous *NPC1* mutated patients and probably evaluated as a potential disease‐modifying treatment for AD.

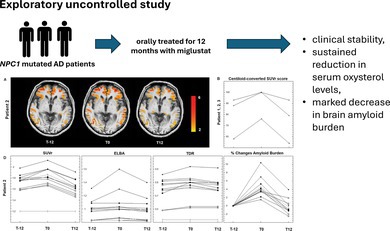

## 1 Introduction

The estimated number of people worldwide affected by dementia was more than 55 million in 2019; for 2050 some sources predict more than 150 million cases globally [1]. Alzheimer's disease (AD) is the leading cause of dementia, accounting for 60% to 80% of cases and afflicting about 24 million people worldwide [2]. A very small subgroup of AD is represented by autosomal dominant AD (ADAD), mainly related to mutations in *PSEN1*, *PSEN2* and *APP*, which interfere with the physiological processing of the Aβ peptide [3]. The pathological features of ADAD mirror those of the more common sporadic form.

Due to the severity, social impact, and increasing prevalence of AD in the population, the need for effective treatments becomes more and more urgent. However, a profound understanding of AD pathogenesis is necessary to develop disease‐modifying therapies. In recent years, comprehensive hypotheses regarding a primarily lipid pathogenesis of AD have emerged [4, 5]; these complex models depict AD as a lipid disorder where altered cholesterol homeostasis, leading to increased intracellular cholesterol levels, plays a key pathogenetic role and precedes the so‐called “amyloid cascade”.

Notably, late‐onset neurodegenerative diseases including AD, Parkinson's disease, or multiple system atrophy are relatively common within Niemann Pick disease Type C (NPC) families [6]. According to this observation, individuals with heterozygous *NPC1* mutations have been found to have an increased risk of parkinsonism or dementia [7], and several studies have discovered monoallelic *NPC1* mutations in cohorts of patients with dementia and brain amyloid deposition [8, 9]. NPC is caused by biallelic mutations in *NPC1* or *NPC2*, both of wich play a role in the turnover of lipids, particularly cholesterol, in the endolysosomal system [10]. Thus, mutation carriers show impaired cell sterol trafficking, resulting in the accumulation of free cholesterol within the lysosomal and late‐endosomal compartment [11]. The possible association between heterozygous *NPC1* mutations and dementia was also strongly suggested by a mouse model: aged *NPC1*+/− mouse brains showed cholesterol accumulation, Purkinje cell loss and enhanced tau phosphorylation [12].

Thus, AD and NPC represent two distinct diseases sharing some disease‐related molecular pathways, including cholesterol metabolism defects but also involvement of amyloid‐β (Aβ) and tau pathology [13].

In a recent study, we identified the first family with ADAD caused by a novel heterozygous *NPC1* mutation [14]. This novel disease association, combined with the growing evidence supporting a lipid‐based pathogenesis of AD, suggests new therapeutic possibilities for AD. Miglustat is a drug currently used for the treatment of NPC1, a severe disease caused by biallelic *NPC1* mutations. Miglustat impacts the metabolism of oxysterols and has been shown to exert an anti‐amyloidogenic effect in a human cellular model of AD [ 15] and reverts impairment of long‐term potentiation in a mouse model of NPC [16]. Indeed, Miglustat was able to regulate Aβ generation and slightly increase sAPPα secretion by modifying the proportions of various sphingolipid species [15].

In this report, we share our experience using miglustat in three *NPC1*‐mutated siblings with AD from the aforementioned family.

## 2 Methods

### 2.1 Study Medication and Design

Miglustat (1,5 (butylimino)‐1,5‐dideoxy‐D‐glucitol) is a synthetic derivative of a family of polyhydroxylated alkaloids or imino sugars that inhibits glucosylceramide synthase, an essential enzyme for the synthesis of most glycosphingolipids [17]. Miglustat was approved by the Food and Drug Administration for the treatment of Gaucher's disease [18] and also licensed in the European Union for patients with NPC [19]. The recommended dose for adult NPC patients is 200 mg three times a day. Miglustat is known for its ease of use, oral administration, and ability to cross the blood–brain barrier. The most common side effects include gastrointestinal disturbances which are typically transitory and can be managed with dietary adjustments and probiotics. However, no experiences have been reported in patients over 70 years of age so far [20].

In the present study, we administered miglustat to three late‐onset AD patients. They shared the heterozygous c.3034G>T (p.Gly1012Cys) mutation in *NPC1* (NM_000271.5, rs1555632941) [14] and met the criteria for the diagnosis of AD according to the revised criteria for diagnosis and staging [21], as well as the ATN classification system [22], following CSF sampling and amyloid‐PET imaging. However, based on the NPC suspicion index, they did not meet the criteria for the diagnosis of NPC; moreover, aside from the heterozygous p.Gly1012Cys mutation in *NPC1*, whole exome sequencing did not detect any other pathogenic or likely pathogenic variants that could be correlated with the patients' phenotype. Additionally, no deletions or duplications were found in *NPC1, NPC2* and *SMPD1* [14]. We excluded from the study other *NPC1* mutated AD patients from the same family due to their struggles with oral therapy and subsequent poor compliance.

The therapeutic use of miglustat in our patients was approved by the local ethics committee (protocol n° 23, 314/2023). Informed consent was obtained from the patients or their legal representatives. Miglustat was orally administered, starting at a dose of 200 mg per day. After one week, the dosage was increased to 400 mg per day. After two weeks miglustat was further increased to 600 mg per day. The patients continued with 600 mg per day divided into three daily doses for 12 months. The drugs they were taking before were not changed during this period. They underwent monthly clinical evaluations and routine blood tests. Neuropsychological evaluations were conducted at baseline (T0), 6 months (T6), and 12 months (T12). Brain amyloid‐PET imaging was performed at T0 and T12. Serum oxysterol concentrations were analyzed at T0, T3, T9, and T12. CSF neurodegeneration biomarkers and oxysterol concentrations were measured at T12. We also compared the data obtained from cognitive tests, brain amyloid PET scans, and biochemical analyses on plasma and CSF with those observed 1 year before treatment (T‐12) to better outline their trend over time.

### 2.2 Biochemical Analyses

Sterols and oxysterols were quantified using isotope dilution‐gas chromatography–mass spectrometry on plasma and CSF samples collected from the three patients and 45 healthy age‐matched controls (mean age 75.5 ± 3.1 years old, 22 males and 23 females) as previously described [14, 23]. We quantified lathosterol, desmosterol, 7β‐hydroxycholesterol (7βOHC), 7‐ketocholesterol (7‐KC), 5β,6β‐epoxy cholesterol, 5α6α‐epoxy cholesterol, and cholestane‐3β,5α,6β‐triol (C‐triol).

CSF markers of AD pathology (amyloid‐β 40 and 42 [Aβ40 and Aβ42], total‐tau [tTau], and tau phosphorylated at amino acid 181 [pTau181]) were analyzed using Lumipulse assays as previously described [24].

### 2.3 Amyloid‐PET Imaging

All patients underwent [18F] flutemetamol—a radioligand‐based amyloid‐β (Aβ) PET‐CT scans using a fully automated multiparametric PET acquisition protocol (Flow Motion Multiparametric PET, Siemens Biograph Vision 600 PET/CT scanner, Siemens Healthineers, Knoxville, USA) with a 26.2‐cm axial field‐of‐view. PET images were reconstructed using PET software version VG76A and list‐mode data from a dual time‐window protocol (0–10 min and 90–120 min) after radioligand injection (reconstruction parameters: TrueX +TOF, 4 iterations, 5 subsets, 440 matrices, 1‐mm Gaussian filter and relative correction for point spread function, scatter and attenuation from non‐contrast‐enhanced CT with 130 kV, 25 mAs, 1.5 mm slice spacing and pitch 0.75, CareDose4D, CarekV, Admire level 3; 3–5 mm full‐width half‐maximum on transaxial images) [25]. Visual and semiquantitative measurements of neocortical Aβ binding in vivo were performed on amyloid PET scans using three independent approaches which were proven to accurately rank the brain amyloid burden; for example, the standardized uptake value ratio (SUVr), ELBA (based on radiomic features) and time‐delayed ratio (TDr, which targets the area of maximum perfusion) [26]. Longitudinal measurements of Aβ binding were thus performed to evaluate time‐dependent changes in amyloid burden in the whole brain (e.g., whole brain SUVr, ELBA and TDr) and in a set of cortical regions (e.g., frontal, occipital, posterior parietal, lateral temporal, precuneus/posterior cingulate SUVr, ELBA and TDr). For this purpose, results of each amyloid analysis for each patient were compared with a validated database of healthy age‐matched controls. The overall rank parameter namely the gray matter compound score derived from each independent whole‐brain semiquantitative measurement (range 0–1, cut‐off for a positive scan: > 0.5) and the patient centiloid‐converted SUVr score were calculated to obtain the likelihood for a positive or negative increase in amyloid burden. Percentage changes of whole brain and cortical Aβ binding were calculated [27].

### 2.4 Subjects and Pre‐Treatment Evaluation

**FIGURE 1:**
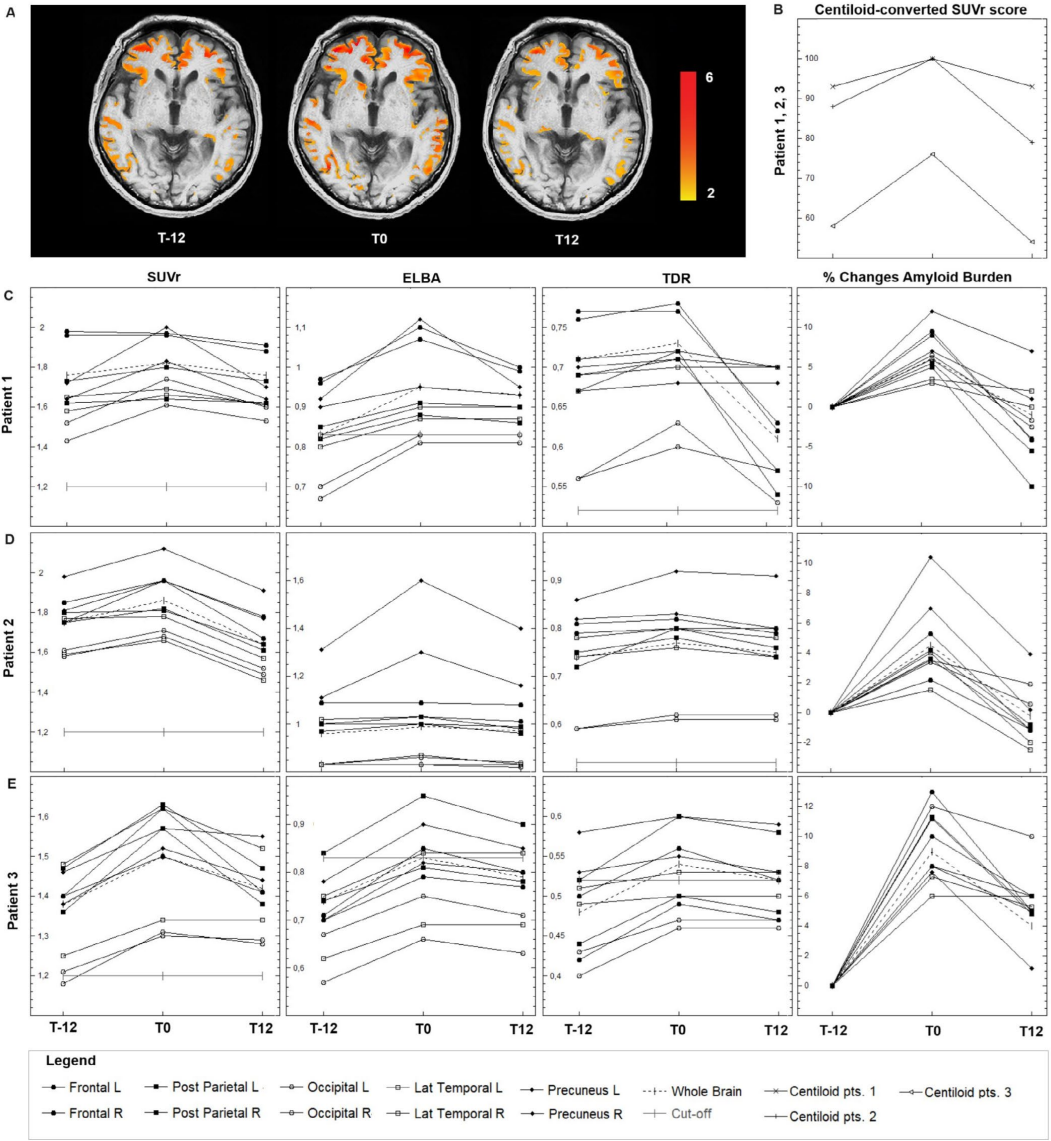
(A) Cortical amyloid burden of patient 2 superimposed on co‐registered T1‐weighted structural MRI images in the pre‐treatment (T‐12), starting treatment (T0) and post‐treatment (T12) conditions. We isolated the cerebral structures using a segmentation technique (FMRIB's Software Library, FSL, http://www.fmrib.ox.ac.uk/fsl) on the MRI scan into gray matter (GM), white matter and cerebrospinal fluid. Cortical amyloid burden was extracted by using the segmented GM‐MRI mask. (B) Time‐dependent changes of centiloid‐converted SUVr scores from T‐12 to T0 and T12 in patients 1, 2, and 3. (C–E) Time‐dependent changes of SUVr, ELBA, and TDr in the whole brain and in a set of cortical regions (e.g., frontal, occipital, posterior parietal, lateral temporal, precuneus/posterior cingulate) and percentage changes (pooled data) of whole brain and cortical Aβ binding.

**FIGURE 2:**
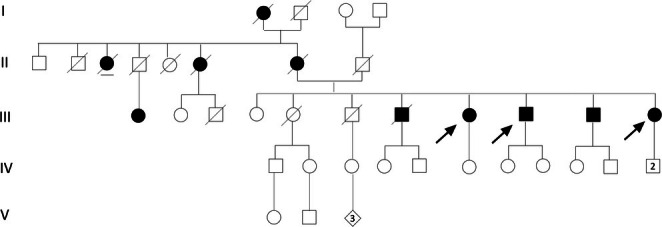
Pedigree of the family. The described patients are indicated with arrows: patient 1 (III;8), patient 2 (III;9), patient 3 (III;11). The mother (II;7) had memory loss from age 61 and then she was diagnosed with AD. AD was diagnosed in a deceased brother (III;7) with onset at 56 years, in a brother (III;10) with onset in his sixties, in two maternal aunts (II;3 and II;6), the daughter of a maternal uncle (III;1) and the maternal grandmother (I;1).

The enrolled subjects belong to a large, three‐generation family with ADAD [14]. We have already detailed most of the data reported here in our previous paper [14] as pre‐treatment (T‐12) evaluation. This includes neuropsychological tests, CSF markers of amyloid deposition, tau pathology and neurodegeneration (ATN), brain amyloid‐PET, and serum oxysterols. All our three patients met the criteria for a diagnosis of AD according to the revised guidelines for diagnosis and staging [21] and the ATN classification system [22]. Below is a summary of the main findings, mostly available in Tables 1, 2, 3 and Figures 1 and 2.

**TABLE 1:** Neuropsychological test scores adjusted for age and educational level.

Months	MMSE (normal ≥ 23.8)	MODA (normal > 85.5)	CDT (normal > 6)	15 words Rey immediate recall (normal ≥ 28.53)	15 words Rey delayed recall (normal ≥ 4.69)
P1					
T‐12	14	65.6	/	/	/
T0	11	47.5	2	/	/
T6	9	/	0	/	/
T12	9	/	0	/	/
P2					
T‐12	23.4	84.4	9	/	/
T0	17	74.9	9	/	/
T6	17	76.4	7	/	/
T12	17	74.9	9	/	/
P3					
T‐12	28.05	/	9	/	/
T0	29.15	/	8	38	6.24
T6	26.15	/	6	28.22	5.48
T12	26.15	/	7	28.22	5.48

**TABLE 2:** Summary of CSF and plasma findings.

Months	Triol plasma μg/L (n.v. 15.46 ± 8.88)	7KC plasma μg/L (n.v. 31.03 ± 8.43)	Triol CSF μg/L (n.v. 0.7 ± 0.1)	7KC CSF μg/L (n.v. 0.86 ± 0.25)	Aβ42 CSF pg/mL (normal > 450)	Aβ 42/40 CSF (normal > 0.060)	Tau CSF pg/mL (normal < 275)	pTau CSF pg/mL (normal < 55)
P1								
T‐12	63.6	105.36	1.19	1.38	471	0.051	747	94
T0	59.08	110.08	/	/	/	/	/	/
T3	42.86	89.22	/	/	/	/	/	/
T9	30.84	62.58	/	/	/	/	/	/
T12	26.6	63.81	/	/	/	/	/	/
P2								
T‐12	58.16	135.32	1.28	1.18	271	0.027	1520	329
T0	47.16	128.9	/	/	/	/	/	/
T3	42.81	102.5	/	/	/	/	/	/
T9	44.47	98.54	/	/	/	/	/	/
T12	40.43	99.68	0.84	0.95	776	0.036	1705	300
P3								
T‐12	32.16	119.12	0.94	2.12	428	0.053	486	58
T0	39.06	120.38	/	/	/	/	/	/
T3	32.07	81.88	/	/	/	/	/	/
T9	26.81	68.12	/	/	/	/	/	/
T12	24.15	60.08	0.67	1.19	961	0.057	623	90

**TABLE 3:** Clinical description and brain amyloid‐PET at T‐12.

	Diagnosis	Age/age of onset	*APOE*	MMSE/MODA	Amyloid accumulation at brain amyloid‐PET	Suspicion index (criteria met) [15]
P1	AD	77/70	ε3/ε3	14/30–65.6	+++ (frontal, lateral temporal, posterior parietal, occipital, precuneus and posterior cingulate)	0
P2	AD	75/69	ε3/ε3	23.4/30–84.4	+++ (lateral temporal, prefrontal, posterior parietal, precuneus and posterior cingulated cortex)	20 (psychotic symptoms, treatment‐resistant psychiatric symptoms, other psychiatric disorders)
P3	AD	69/69	ε3/ε3	28.05/30 —	+ (lateral temporal, posterior parietal and occipital cortex)	5 (other psychiatric disorders)

Abbreviations: AD, Alzheimer's disease; MMSE, Mini Mental State Examination; MODA, Milan Overall Dementia Assessment; n.a., not available.

Patient 1 (patient 1 in the previous study [14]), a female aged 77 at T0, showed a moderate to marked cognitive decline that began at age 70 with memory disturbances and slowly worsened with a progressive reduction in daily activities autonomy. Patient 2 (patient 2 in the previous study [14]), a male aged 75 at T0, had a mild to moderate cognitive impairment that started at age 69 with memory and attention issues and progressed over time. At the age of 74 he started experiencing psychiatric disturbances including depression, visual hallucinations, and impulse control disorder, which required neuroleptic treatment. Patient 3 (patient 4 in the previous study [14]), a female aged 69 at T0, presented short‐term memory disturbances, deficits in construction praxis skills, and mild mood depression. Despite these challenges, she had preserved general cognitive functions.

In all three patients, CSF markers of neurodegeneration showed a typical AD pattern (A+, T+, N+). Amyloid‐PET indicated brain amyloid deposition, marked in patients 1 and 2 and moderate in patient 3. Thus, they clearly showed abnormalities on specific Core 1 biomarkers according to the most recent diagnostic criteria for AD [ 21]. Assessment of oxysterols showed a disrupted profile in all subjects, particularly with increased levels of 7KC and C‐triol in both serum and CSF. Regarding AD therapy, patients 1 and 2 were taking anticholinesterase drugs (rivastigmine 6 mg twice daily) and memantine (20 mg daily) since diagnosis; patient 3 was untreated. None of the patients had previously received treatment with anti‐amyloid monoclonal antibodies or been included in clinical trials. The three patients were carriers of the Apo E3/E3 haplotype. To explore the eventual presence of NPC‐associated features, we used the Suspicion Index, a well‐recognized tool with high discriminatory performance with cutpoints for grading suspicion of NPC [28]. All the patients scored a total risk prediction of < 40 (respectively 0, 20, and 5); notably, a score of ≥ 70 highly suggests a possible NPC diagnosis [29].

## 3 Results

The patients took miglustat regularly, which was generally well tolerated. However, a slowing down in the dose increase was necessary in the first 2 months of therapy due to diarrhea, especially in patient 1. Another side effect was a slight weight loss, possibly related to the gastrointestinal disturbances. Accordingly, a tailored diet and a probiotic (saccharomyces boulardii) were recommended. No alterations worth mentioning were found during the routine laboratory exams.

Neuropsychological tests showed a substantial stabilization of cognitive performance from T0 to T12 in patient 2. In patients 1 and 3, the cognitive skills initially continued to worsen, as demonstrated by the evaluation at T6; however, in the following 6 months, a clinical stabilization was achieved, as confirmed by the evaluation at T12 showing unchanged cognitive functions. The scores of neuropsychological evaluations, which were conducted on each occasion by the same clinical neuropsychologist (B.P.), are detailed in Table 1.

Biochemical analyses were conducted on both serum and CSF, and the values were compared to those of healthy matched controls. Unfortunately, Patient 1 declined consent for a repeat lumbar puncture, so only the pre‐treatment CSF sample was available for this subject. Serum oxysterol assessment at T0 showed very high levels of 7KC and C‐triol, similar to those observed a year earlier. Throughout treatment with miglustat we noted a persistent decrease in both 7KC and C‐triol plasma levels. In parallel, 7‐KC and C‐triol CSF levels decreased at T12 compared to the values measured one year before starting therapy (T‐12).

Of particular interest, analysis of ATN biomarkers in CSF revealed a marked increase in Aβ42 amyloid and an increased Aβ42/40 ratio at T12 compared to pre‐treatment values (T‐12). Conversely, tTau levels increased and pTau181 remained stable or increased at T12 compared to T‐12. Detailed plasma and CSF findings are outlined in Table 2. Furthermore, Tables S1 and S2 show the values of other oxysterols measured in plasma and CSF.

Brain amyloid‐PET visual and semiquantitative analyses showed a pathological amyloid burden in the whole brain and in each cortical region in all three of our subjects at pre‐treatment evaluation (T‐12). Indeed, considering the centiloid thresholds established in literature and used in clinical trials [30], centiloid values were consistent with an elevated AD‐typical cortical Aβ deposition in the frontal, temporal, posterior parietal, precuneus and posterior cingulate regions.

Amyloid‐PET performed at T0 revealed a further increase in amyloid deposition in all of them, particularly evident in patient 2, and in the frontal, precuneus/posterior cingulate and temporal cortices. Surprisingly, brain amyloid burden decreased at T12 in all three patients. The reduction of neocortical Aβ binding was mainly evident in patients 2 and 3, particularly in the frontal and precuneus/posterior cingulate cortices. Figure 1 details the brain amyloid‐PET findings and their changes over time in our three patients.

## 4 Discussion

The relationship between the cholesterol pathway and AD has been scientifically debated for a long time. Notably, in 1906 Alois Alzheimer described not only amyloid plaques and neurofibrillary tangles, but also intracellular lipid deposits [31]. A cholesterol level increase promotes APP processing through the amyloidogenic pathway [32]. Additionally, both β‐ and γ‐secretases exhibit higher activity in cholesterol‐rich lipid rafts. The interaction of Aβ with cholesterol in the plasma membrane and lipid rafts may play a role in Aβ seeding, aggregation, and toxicity [33]. The large amount of findings about this topic has recently given rise to new comprehensive pathogenetic models underlying the key role of altered brain cholesterol metabolism in AD, recently defined as a possible lipid disorder [4, 5].

Bidirectional links between NPC and AD have been previously well described [13]. These two pathologies share intriguing neuropathological similarities, including neurofibrillary tangles, deregulated Aβ metabolism, and altered cholesterol metabolism. In both conditions the lipid imbalance may be a driver in the neurodegenerative process and other similarities also lie on the genes involved in cholesterol metabolism [13]. Altered amyloid metabolism in NPC is influenced by cholesterol accumulation [13], and might be related to the development of pathology [34]. However, the increased CSF Aβ concentration in NPC, although partially unknown, has not been described in any other neurodegenerative disease. Further links between NPC and AD are provided by evidence of lysosomal dysfunction in AD [ 35] and the increase of NPC1 levels in degenerated brain regions of AD patients [36].

To further support the link between the diseases, heterozygous *NPC1*‐mutated subjects have been rarely reported to manifest clinically with parkinsonism or dementia [7]. Additionally, different studies have found heterozygous *NPC1* mutations in cohorts of adults with dementia and brain amyloid deposition [8, 9].

Our group previously described the first family with ADAD due to a heterozygous *NPC1* mutation [14]. The clinical, biochemical, molecular, and brain imaging data in our family, together with the results of several previous studies about the role of heterozygous *NPC1* mutations [8, 9], supported a possible novel entity of ADAD.

In line with previous studies, 7‐KC and plasma oxysterols levels were significantly increased in *NPC1* carriers compared with healthy controls [37, 38], although they were lower compared to NPC patients. Differently from previous studies, that included *NPC1* carriers with an average age of about 40 years [37, 38], our patients' ages range between 68 and 78 years, thus supporting the idea that the most altered biochemical phenotype may be associated with old age. This hypothesis is in line with the mouse model of Yu et al. [12]: only in aged *NPC1*+/− mouse brains but not in young *NPC1*+/− mouse brains, they demonstrated cholesterol accumulation and histopathological signs of neurodegeneration.

This possible novel entity of ADAD, as well as the growing evidence of a lipid‐mediated pathogenesis of AD, prompted us to explore potential therapeutic implications. Indeed, several NPC treatments capable of slowing down disease progression by reducing cellular lipid levels [39] are currently available. Miglustat can inhibit glucosylceramide synthase, thus reducing the brain load of GM2 and GM3 gangliosides in NPC. Mattsson et al. [40] demonstrated that treated NPC patients had lower CSF levels of the Aβ peptide Aβ1‐42, and the APP‐derived peptides sAPP‐α and sAPP‐β, suggesting that treatment may alter amyloid metabolism. Moreover, CSF T‐tau levels decreased after the start of miglustat treatment [40].

Interestingly, several studies reported a significant increase in gangliosides (GM2 and GM3) in the brains of AD patients [41]. Additionally, specific gangliosides may promote Aβ production and/or its assembly into neurotoxic complexes in AD [42]. Interestingly, miglustat, has been shown to have an anti‐amyloidogenic effect in a human cellular model of ad [ 15]. Miglustat was indeed able to regulate Aβ generation at the level of the β‐cleavage. Concurrently, it also tended to slightly increase the sAPPα secretion thus favoring the non‐amyloidogenic pathway. It is likely that miglustat, through strong modifications in the proportions of various sphingolipid species, particularly sphingomyelins and ceramides, is able to modulate the APP processing and Aβ production [15].

Therefore, since miglustat can easily cross the blood–brain barrier and is well tolerated by humans, we speculated its possible use as a disease‐modifying therapy for AD, especially in AD patients harboring a heterozygous *NPC1* mutation.

Here we present for the first time the effects of a therapeutic dose of miglustat in AD by treating three heterozygous *NPC1‐mutated* AD patients from our previously described family [14].

We observed a decrease in serum levels of 7‐KC and C‐triol in all three subjects, which was mirrored by a reduction of 7‐KC and C‐triol also in the CSF in the two patients who underwent a follow‐up lumbar puncture. Although the reduction of plasma and CSF oxysterol levels could have been expected, the brain imaging and CSF results regarding amyloid levels were surprising.

Brain amyloid‐PET analysis in our subjects was consistent with an elevated AD‐typical cortical Aβ deposition in the frontal, temporal, posterior parietal, precuneus and posterior cingulate regions [14]. Although a previous amyloid‐PET study in two NPC patients showed an amyloid burden limited only to the frontal areas [43], another PET study including eight NPC patients and seven healthy controls showed no patient with significant Aβ‐amyloid pathology [44]. These PET findings highly support the association of monoallelic *NPC1* mutations with a novel entity of ADAD.

Brain amyloid‐PET performed after 1 year of treatment with miglustat (T12) showed a sharp decrease in amyloid burden in all the patients, which instead had increased at T0 compared to 1 year earlier (T‐12). This trend over time may reflect disease progression before treatment, followed by a possible slowing of the neurodegenerative process linked to brain amyloid deposition during miglustat therapy.

As for ATN markers in CSF, all three of our patients clearly showed different levels from what was observed in NPC patients. While previous studies showed normal CSF p‐tau levels in NPC patients [45, 46], the increase of p‐tau levels observed in our patients, appears to be rather an AD‐specific feature [47]. As for Aß42, two of our patients showed clearly lower levels than the normal limits, as expected for an AD pattern. Although patient 1 had a value of 471 pg/mL, the Aβ42/40 ratio was lower than the normal limit. On the contrary, NPC patients showed Aβ isoforms Aβ42 and the Aβ42/40 ratio higher than controls, without any correlations with disease duration [45].

Notably, data obtained through the analysis of ATN markers in the CSF suggest the hypothesis that miglustat may reduce brain amyloid load. Specifically, we observed a marked increase in Aβ42 levels and an increased Aβ42/40 ratio in the two available CSF samples at T12 compared to pre‐treatment levels (T‐12). It is possible that this increase is even greater than measured, as values at T0 may have been further reduced compared to pre‐treatment levels (T‐12), especially if we hypothesize a similar trend with amyloid‐PET. A recent study on anti‐Aβ monoclonal antibodies suggests that the increase in CSF Aβ42 could potentially benefit individuals with AD since it is independently associated with slowing cognitive decline and clinical deterioration [48]. On the other hand, we did not observe any effect on tTau and pTau181 levels during miglustat treatment in our patients. Notably, the marked increase of tTau levels at T12 found in our patients contrasts with the reduction of tTau previously reported under treatment with miglustat in NPC1, where miglustat did not produce significant changes in Aβ42 levels [40]. This difference may suggest a possible different mechanism of therapeutic action of miglustat in AD compared to NPC1. However, a larger study involving more patients is needed to validate our preliminary findings.

The clinical course during miglustat treatment showed stabilization in one patient since the introduction of therapy; in the other two subjects cognitive performance continued to worsen over the first 6 months of therapy but stabilized over the next 6 months. This trend could possibly be related to a time‐dependent effect of miglustat: the interaction with cholesterol metabolism leading to a decrease in amyloid burden and clinical stabilization of an already started pathological process may take time, as observed with anti‐amyloid monoclonal antibodies that show a time‐dependent effect on slowing cognitive decline [49].

The reduction of CSF amyloid and PET amyloid burden after miglustat treatment may possibly be linked to a decrease in beta amyloid production through interaction with cholesterol homeostasis, thus enhancing the endogenous clearance of beta amyloid from the brain [50]. Thus, if our findings are confirmed by future clinical trials, the mechanisms of action of miglustat might be synergistic and additive with the anti‐amyloid monoclonal antibodies. In this case, miglustat could potentially act as a maintenance therapy after the initial immunotherapeutic removal of beta amyloid.

However, the present study showed several limitations. It was uncontrolled and the small number of subjects enrolled in the present exploratory study does not allow us to support conclusions of efficacy of Miglustat in the studied condition. Accordingly, biomarker measures used in this study showed a high degree of variability. Although patients 1 and 2 were taking anticholinesterase drugs and memantine at the same doses throughout the study period, and not known effects between standard AD therapies and Miglustat have ever been reported, we cannot completely exclude a possible influence of these treatments in addition to Miglustat on the cognitive performance of patients. Moreover, since all three of our patients belong to the same family, their shared genetic background and environmental factors may have influenced our findings. Additionally, while biochemical findings of the present study were compared to healthy controls, the absence of clinical control groups (e.g., sporadic AD patients or heterozygous *NPC1* carriers without AD) makes it difficult to determine whether the observed effects are specific to *NPC1*‐related AD or more generalizable. However, despite the presence of several limitations, our study allows us to generate hypotheses thus supporting future structured clinical trials.

In conclusion, our data hypothesize for the first time a possible anti‐amyloidogenic effect of miglustat in heterozygous *NPC1‐*mutated AD patients and pave the way for future controlled trials involving a larger number of patients. Our *NPC1*‐mutated subjects likely represent a unique model of AD that may be particularly responsive to miglustat, a drug currently approved for NPC. However, the observed anti‐amyloidogenic effect of miglustat may be even independent of the presence of *NPC1* mutation [15]. Since miglustat could act not only by reducing amyloid deposition but also by exerting a direct effect on lipid‐mediated damage, which plays a key role in AD pathogenesis, we cannot completely exclude future trials to investigate the possible use of miglustat as a disease‐modifying treatment in sporadic classical AD.

## Author Contributions

D.L. and A.M. contributed to the conception and design of the study. D.L., D.G., S.B., B.P., D.T., V.L., A.C., S.S., and A.M. contributed to the acquisition and analysis of data. D.L., N.D., H.Z., and A.M. contributed to drafting the text.

## Ethics Statement

Studies were performed and samples were obtained after local ethics committee approval, in accordance with the Helsinki Declaration of 1964, as revised in October 2013 in Fortaleza, Brazil. Informed consent for molecular genetic analysis and clinical procedures was obtained from the patients or their legal representative.

## Conflicts of Interest

H.Z. has served on scientific advisory boards and/or as a consultant for Abbvie, Acumen, Alector, Alzinova, ALZPath, Amylyx, Annexon, Apellis, Artery Therapeutics, AZTherapies, Cognito Therapeutics, CogRx, Denali, Eisai, LabCorp, Merry Life, Nervgen, Novo Nordisk, Optoceutics, Passage Bio, Pinteon Therapeutics, Prothena, Red Abbey Labs, reMYND, Roche, Samumed, Siemens Healthineers, Triplet Therapeutics, and Wave, has given lectures sponsored by Alzecure, BioArctic, Biogen, Cellectricon, Fujirebio, Lilly, Novo Nordisk, Roche, and WebMD, and is a co‐founder of Brain Biomarker Solutions in Gothenburg AB (BBS), which is a part of the GU Ventures Incubator Program (outside submitted work). None of the other authors declare conflicts of interest.

## Supporting information


**Table S1:** Other oxysterols in plasma measured by isotope dilution GCMS.
**Table S2:** Other oxysterols in CSF measured by isotope dilution GCMS.

## Data Availability

The data that support the findings of this study are available from the corresponding author upon reasonable request.
