# Supplementary material for: Miglustat in Alzheimer's Disease Associated With Heterozygous NPC1 Mutation: Exploratory Case Series and Preliminary Findings
Source: Eur J Neurol. 2025 Nov 11;32(11):e70419. doi: 10.1111/ene.70419 (PMC12603779; doi:10.1111/ene.70419)
Supplement: Supplementary file 1 — Table S1: Other oxysterols in plasma measured by isotope dilution GCMS. Table S2: Other oxysterols in CSF measured by isotope dilution GCMS. [file ENE-32-e70419-s001.docx]

**Supplementary information**

**Supplementary Table 1: Other oxysterols in plasma measured by isotope dilution GCMS**

| **Patient** | **Time point** | **Lathosterol**  **(µg/L, n.v. 1508.96±616.37)** | **Desmosterol**  **(µg/L, n.v. 780.41±371.08)** | **7bOHC**  **(µg/L, n.v. 13.4±5.7)** | **5,6a-epoxy**  **(µg/L, n.v. 28.78±11.46)** | **5,6b-epoxy**  **(µg/L, n.v. 27.32±17.65)** |
| --- | --- | --- | --- | --- | --- | --- |
|  | T-12 | 1095 | n.a. | 11.84 | 71.1 | 51.6 |
| **P1** | T0 | 995.24 | 798.44 | 39.92 | 61.47 | 28.7 |
|  | T3 | 796.64 | 587.52 | 33.42 | 52.45 | 25.11 |
|  | T9 | 762.24 | 608.92 | 15.84 | 48.38 | 26.37 |
|  | T12 | 505.17 | 597.72 | 21.5 | 46.01 | 25.62 |
|  |  |  |  |  |  |  |
|  | T-12 | 492.89 | n.a. | 6.88 | 61.1 | 43.36 |
| **P2** | T0 | 330.72 | 302.52 | 29.3 | 51.92 | 42.22 |
|  | T3 | 233.48 | 265.92 | 13.84 | 48.56 | 37.26 |
|  | T9 | 314.56 | 243.56 | 22.42 | 42.16 | 40.33 |
|  | T12 | 267.48 | 220.32 | 24.43 | 45.7 | 38.67 |
|  |  |  |  |  |  |  |
|  | T-12 | 897.6 | n.a. | 16.48 | 95.86 | 94.86 |
| **P3** | T0 | 1386.32 | 863.28 | 43.12 | 60.07 | 39.81 |
|  | T3 | 1237.8 | 687.36 | 41 | 45.39 | 31.41 |
|  | T9 | 984.48 | 616.8 | 30.88 | 40.68 | 28.54 |
|  | T12 | 1004.76 | 661.2 | 24.28 | 42.83 | 30.42 |

**Supplementary Table 2: Other oxysterols in CSF measured by isotope dilution GCMS**

| **Patient** | **Time point** | **Lathosterol (ng/L)** | **Desmosterol (ng/L)** | **7bOHC (µg/L)** | **5,6a-epoxy (µg/L)** | **5,6b-epoxy (µg/L)** |
| --- | --- | --- | --- | --- | --- | --- |
| **P1** | **T-12** | 1534.2 | 809.31 | 4.64 | 5.12 | 1.67 |
|  | **T12** | n.a. | n.a. | n.a. | n.a. | n.a. |
| **P2** | **T-12** | 1814.24 | 936.02 | 5.62 | 7.03 | 1.91 |
|  | **T12** | 1706.11 | 843.32 | 5.71 | 6.14 | 1.03 |
| **P3** | **T-12** | 2201.15 | 1047.79 | 4.63 | 6.95 | 1.76 |
|  | **T12** | 1984.34 | 918.64 | 4.05 | 5.85 | 1.12 |
